# Tropical Diabetic Hand Syndrome: report of 2 cases

**Published:** 2012-06-06

**Authors:** Ngim Ewezu Ngim, Paul Amah, Innocent Abang

**Affiliations:** 1 University of Calabar Teaching Hospital, Calabar, Nigeria

**Keywords:** Tropical, diabetic, hand, syndrome

## Abstract

Diabetes mellitus is known to be complicated by foot lesions. However, a few cases of hand complications have been reported in some parts of the world. The case notes of two patients who presented with this condition in our hospital were retrieved and relevant information extracted. The cases of diabetic hand complications seen in our centre are here reported to draw the attention of medical practitioners to the occurrence of this potentially debilitating and occasionally fatal condition. Appropriate counselling of diabetic patients on hand care strategies and good glycemic control are important preventive measures.

## Introduction

Diabetes mellitus is a metabolic disorder in which there is hyperglycaemia as a result of quantitative and/or qualitative deficiency of insulin. The resulting derangement in glucose metabolism has far reaching consequences on various organ systems in the body. Though primarily a medical condition, some of the complications of diabetes mellitus affecting the limbs may warrant the involvement of the Orthopaedic Surgeon in the management of the patients. The most commonly described lesion affecting the extremity is the diabetic foot syndrome whose manifestation ranges from cellulitis, ulceration to gangrene. However, of recent similar lesions affecting the foot in diabetics are being seen in the hand of these patients. The first of these hand lesions was described in the United States in 1977 [1] and in Nigeria in 1984 [2]. Its incidence seems to be on the rise especially in Africa and India where it is now being referred to as Tropical Diabetic Hand Syndrome (TDHS). Even though this condition in Africa was first described in Nigeria over twenty six years ago [1], not many of such cases have been seen or reported since then. We report two recent cases seen in our centre to draw attention of medical practitioners to this condition that is threatening the function and survival of the hand in diabetic patients in our country.

## Patients and observations

### Patient 1

Mr BL, 45 years old male, lecturer, married, consulted for swelling and pain left middle finger x 3/52 The patient is a known diabetic and hypertensive diagnosed 3years ago but poorly controlled. He noticed a swelling of his left middle finger which gradually increased in size extending to the palm. There was associated pain in the affected finger which was severe, continuous, throbbing and non-radiating, relieved by elevating the hand and analgesics, no known aggravating factors. There was no antecedent history of trauma. The finger gradually became black in colour with foul-smelling discharge and no sensation at the tip. He smokes cigarettes and drinks alcohol regularly. General physical examination revealed a healthy young man, not in any obvious distress, not pale, anicteric. Examination of the left hand showed a dark coloured middle finger, with swelling extending to the palm, tenderness and sensation was found to be absent. Investigations conducted included fasting Blood Sugar which was 15.6mmol/L; urinalysis showed presence of sugar, no ketone bodies; electrolytes, urea and creatinine were within normal range; X-ray showed no bony lesions; Wound swab culture yielded mixed growth of Staphylococcus aureus, coliforms and enterobacteria. The diagnosis of Left Diabetic Hand syndrome was made. The patient had ray amputation of the left middle finger with debridement of the palm. About 50 ml of pus was drained from the hand. Post operative treatment included antibiotics and wound dressings. Secondary closure of the wound was later carried out. Patient recovered satisfactorily and was discharged to the outpatient clinic for follow up. He was also counselled on appropriate care of his hands-prevention of hand injury including the need for use of protective hand devices, seeking prompt and appropriate medical care should any form of hand injury, no matter how minor, occur and care during manicure.

### Patient 2

**Figure 1:**
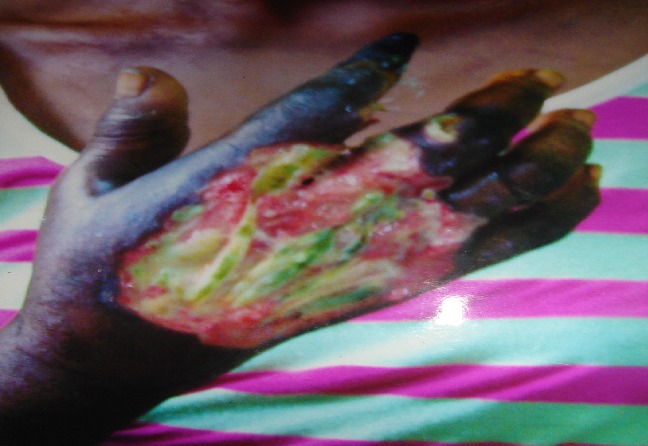
Right hand of Patient 2 showing gangrene of the index finger and ulcer on the dorsum

Madam ME, 60 years old female, married, trader, condulted for painful swelling of the right hand x1/12. Patient noticed a painful swelling on her right index finger which was initially treated as Whitlow. There was no history of trauma. The swelling later progressed to involve other fingers and the dorsum of the hand. There was associated fever and pain which was relieved by analgesics and elevation. She became unconscious 10hours before presentation in hospital. She was not a known hypertensive. General Physical Examination revealed an ill-looking woman, afebrile, not pale. Examination of the right hand revealed a swollen and tender with discharge from the palmar surface, herbal dressings over the wound, fluctuant, differential warmth+, black index finger, sensation was equivocal. Radial pulse was palpable (Figure 1). Urinalysis revealed sugar and ketone bodies in urine; Fasting blood sugar = 17 mmol/L; X-ray showed no bony lesions, Wound swab for culture yielded Coliforms. The diagnoses of diabetic ketoacidosis (DKA) and right diabetic hand syndrome were made. The DKA was appropriately treated by the medical team and recovered consciousness. Then incision and drainage of the abscess and ray amputation of the index finger were done. She was scheduled for skin cover by the Plastic and Reconstructive Surgeon.

## Discussion

Patients with diabetes mellitus have impaired immunologic responses to combat infections [3]. Infection and ulceration of the hand is a major cause of morbidity and mortality in certain populations in Africa. Hand ulceration and infection in diabetic patients was first described in the United States in 1977 [1] and in Africa in 1984 [2]. Subsequently, the majority of reported cases have been from various parts of the African continent. The term "Tropical Diabetic Hand Syndrome" (TDHS) has been used to describe specific complication of the hand affecting patients with diabetes mellitus in the tropics.

The syndrome encompasses localized cellulitis with variable swelling and ulceration of the hands, to progressive, fulminant hand sepsis and gangrene affecting the hand or entire limb [4]. It is poorly understood both by patients and clinicians, and severe in consequence without prompt and aggressive treatment.

Patients with TDHS have poorly controlled blood glucose levels but neither peripheral vascular disease nor peripheral neuropathy appear to play a substantial role in the pathogenesis of TDHS [5]. TDHS is less well recognized than foot infections [4]. More recently, TDHS has been reported among patients in India. These data suggest that TDHS occurs primarily in diabetic patients who live in tropical or coastal areas and might result in loss of hand function, amputation, or death [5]. Presentation to hospital is often delayed due to the patients′ unawareness of the potential risks, lack of concern because the initiating trauma might have been trivial, or decision to seek initial help from traditional healers.

In one of the series of TDHS with 25 patients, it was noted that a history of trauma played a role in only 16% of patients, and other studies show either a relatively low incidence of trauma or minimal severity of the antecedent trauma [6]. There was no history of trauma in the two cases being reported. In addition, intra operative cultures yielded multiple organisms in 55% of specimens, gram-negative organisms in 73%, and Staphylococcus aureus in only 36%, a pattern of fora also mirrored in other series [7, 8]. This representative case series suggests that all diabetic hand infections should be treated with caution and the possibility of poly microbial infection should be considered when determining an initial approach to antibiotic coverage.

TDHS can develop into a rapidly progressive, synergistic gangrene (Meleney′s gangrene) confined to the superficial fascia that can result in death within days of onset of symptoms [3, 6]. Although the majority of patients survive, permanent disability is likely. The most common cause of polymicrobial synergistic gangrene is a symbiotic relationship of aerobic gram-negative rods in combination with different enteric anaerobes [6]. Culture of tissue biopsy specimens yields a single bacterial species in >75% of cases, whereas swab cultures yield polymicrobial flora in the majority of cases, probably because of contamination [6]. Therefore, routine swabs of open, infected hands cannot guide optimal antimicrobial therapy and might not be appropriate use of resources in hospitals with limited laboratory facilities.

Independent risk factors for TDHS include poorly controlled diabetes, neuropathy, insulin treatment or malnutrition. Clinicians should be aware of these complications and be prepared to immediately admit TDHS patients to hospital for aggressive surgical intervention (i.e. debridement, pus drainage or amputation) and high-dose, intravenous, broad-spectrum antibacterial therapy that includes anti-anaerobic activity. Without prompt, aggressive treatment TDHS can lead to permanent disability, limb amputation (13% of TDHS patients require major upper limb amputation), or death [4].

Prevention strategies include patient and staff education that focuses on proper hand care, nutrition, and the importance of seeking medical attention immediately following hand trauma regardless of the severity of the injury, or at the earliest onset of hand-related symptoms, such as redness or swelling. Prevention of permanent disability and death due to TDHS will require early recognition by patients and medical practitioners, improved management of glycemic levels in resource-limited countries, and surgical intervention during less severe stages of the condition [4, 5].

## Conclusion

Since this syndrome is less well recognized, it is often under-reported [9]. This necessitated the index report in order to bring to the notice of medical practitioners the existence of this potentially very dangerous hand complication of diabetes mellitus especially in view of the crucial role of early detection in successful treatment.
